# T0901317, an Agonist of Liver X Receptors, Attenuates Neuronal Apoptosis in Early Brain Injury after Subarachnoid Hemorrhage in Rats via Liver X Receptors/Interferon Regulatory Factor/P53 Upregulated Modulator of Apoptosis/Dynamin-1-Like Protein Pathway

**DOI:** 10.1155/2021/8849131

**Published:** 2021-05-28

**Authors:** Jiaxing Dai, Shancai Xu, Takeshi Okada, Yu Liu, Gang Zuo, Jiping Tang, John H. Zhang, Huaizhang Shi

**Affiliations:** ^1^Department of Neurosurgery, The First Affiliated Hospital of Harbin Medical University, Harbin, Heilongjiang, China; ^2^Department of Physiology and Pharmacology, Loma Linda University, Loma Linda, CA 92354, USA; ^3^Department of Neurosurgery, Loma Linda University, Loma Linda, CA 92354, USA; ^4^Department of Anesthesiology, Loma Linda University, Loma Linda, CA 92354, USA

## Abstract

**Methods:**

Subarachnoid hemorrhage (SAH) models of Sprague-Dawley rats were established with perforation method. T0901317 was injected intraperitoneally 1-hour post-SAH. GSK2033, an inhibitor of LXRs, and interferon regulatory factor (IRF-1) CRISPR activation were injected intracerebroventricularly to evaluate potential signaling pathway. The severity of SAH, neurobehavior test in both short- and long-term and apoptosis was measured with Western blot and immunofluorescence staining.

**Results:**

Expression of LXR-*α* and IRF-1 increased and peaked at 24 h post-SAH, while LXR-*β* remained unaffected in SAH+vehicle group compared with Sham group. Post-SAH T0901317 treatment attenuated neuronal impairments in both short- and long-term and decreased neuronal apoptosis, the expression of IRF-1, P53 upregulated modulator of apoptosis (PUMA), dynamin-1-like protein (Drp1), Bcl-2-associated X protein (Bax) and cleaved caspase-3, and increasing B-cell lymphoma 2 (Bcl-2) at 24 h from modeling. GSK2033 inhibited LXRs and reversed T0901317's neuroprotective effects. IRF-1 CRISPR activation upregulated the expression of IRF-1 and abolished the treatment effects of T0901317.

**Conclusion:**

T0901317 attenuated neuronal apoptosis via LXRs/IRF-1/PUMA/Drp1 pathway in SAH rats.

## 1. Introduction

Aneurysmal subarachnoid hemorrhage (SAH) is a severe subtype of stroke affecting patients with poor prognosis such as high mortality within the initial days to weeks after SAH, long-term disability, and cognitive deficits [1, 2]. Even though the early diagnosis and management (clipping or coiling) have improved, we still cannot get the best patient outcome due to early brain injury (EBI) [3]. Apoptosis plays an important role in this pathology process in EBI after SAH [4]. Therefore, targeting neuronal apoptosis is possibly quite important in improving SAH patients' poor prognosis.

Liver X receptors (LXRs) belong to a large family of nuclear receptors which bind to the regulatory region of target genes and, upon ligand binding, stimulate their transcription [5]. LXRs have two subtypes, LXR-*α* and LXR-*β*, sharing 80% identity in their DNA and ligand binding domain amino acid sequences [5, 6]. Both LXR isoforms were detected in central nervous system (CNS) [7]. It was reported that transcriptional action of LXRs is protective in brain injury and the potential use of LXR agonists as therapeutic agents in stroke [8]. Activation of LXRs also provided a potential therapy in Alzheimer disease (AD), Parkinson disease (PD), intracranial hemorrhage (ICH), and other CNS diseases [6, 9–12]. T0901317 is a synthetic agonist of LXRs has been shown to be protective in different models [8, 13]. The LXR agonist TO901317 had potent antiapoptotic effects in acute lung injury, possibly because that decreasing Bax/Bcl-2 ratio and mitochondrial transmembrane potential leaded the release of proapoptotic protein from mitochondrial into the cytosol ultimately resulting in apoptosis [14]. However, the effects of activating LXRs with T0901317 have never been researched in EBI after SAH.

Interferon regulatory factor-1 (IRF-1) is a transcription factor involving in multiple functions including programmed cell death [15, 16]. IRF-1 immunoreactivity was present in 24-72 h after middle cerebral artery occlusion (MCAO) mice and ischemia stroke patients [15]. However, the effects of IRF-1 have never been researched in SAH. It was also reported that LXR activation inhibited neither signal transducer and activator of transcription 1 (STAT1) nor STAT1 translocation to the nucleus but prevented STAT1 from binding to promoters and expression of IRF-1 [17]. IRF-1 binds to distinct sites in the promoter of P53 upregulated modulator of apoptosis (PUMA) and activates PUMA transcription with dynamin-1-like protein (Drp1) accumulations in mitochondria [18, 19].

This research is aimed at revealing antiapoptotic effects of T0901317 via the potential downstream signaling LXRs and IRF-1 in SAH.

## 2. Materials and Methods

### 2.1. Animals

All the experiments were done based on the protocols proposed by the Institutional Animal Care and Use Committee (LACUC) in Loma Linda University. The procedures were performed based on NIH guidelines. We used 300-gram male Sprague-Dawley (SD) rats to establish SAH model. Animals were kept in a comfortable environment with room temperature (22 ± 1°C) and 12/12-hour day/night cycle (humidity: 60 ± 5%). The animals were free to access to food and water.

### 2.2. SAH Model

The endovascular perforation was performed to establish SAH model as previously described [20]. Briefly, animals were anesthetized with 5% isoflurane in 65/35% medical air/oxygen. After anesthesia satisfaction, animals were intubated and then connected to the respirator and an isoflurane vaporizer in the supine position. Left internal and external carotid artery was carefully separated. Two minivessel clips were used to block common carotid artery and internal carotid artery temporarily. A sharpened 4-0 nylon suture was inserted from the cut of external carotid artery to the internal carotid. The suture was advanced further; the depth was about 3.0 cm and withdrawal immediately. In Sham animals, the same procedures were performed except perforation with suture.

### 2.3. Drug Administration

T0901317 was purchased from ApexBio Technology (TX, USA) and dissolved in 30% dimethyl sulfoxide (DMSO). The treatment was administrated intraperitoneally (i.p.) 1 h after SAH. GSK2033 (Sigma, MO, USA) was diluted in DMSO before intracerebroventricular (i.c.v.) injection and administered 1 h before SAH. The control groups received an equal volume of solvents, respectively. IRF-1 CRISPR activation plasmid and control CRISPR were purchased from Santa Cruz (TX, USA) and were injected i.c.v. at 48 h before SAH.

### 2.4. Experimental Designs

*Experiment 1*: 36 SD rats (*n* = 6 per group) were randomly divided into six groups: Sham, SAH 3, 6, 12, 24, and 72 h. In addition, 2 Sham rats and 2 SAH rats were used for immunofluorescence staining. Western blot was used to detect the expression of LXR-*α*, LXR-*β*, and IRF-1. Immunofluorescence staining was performed to check the localization of LXR-*α*, LXR-*β*, and IRF-1 in neurons post-SAH.

*Experiment 2*: to evaluate the short-term outcome, 30 SD rats (*n* = 6 per group) were randomly divided into five groups: Sham, SAH+vehicle, SAH+T0901317 (10 mg/kg), SAH+T0901317 (30 mg/kg), and SAH+T0901317 (90 mg/kg). Based on neurological tests, 30 mg/kg was chosen as the best dosage in the following experiments. Additionally, 12 SD rats (*n* = 4 per group) were randomly divided in 3 groups: Sham, SAH+vehicle, and SAH+T0901317 (30 mg/kg). Immunofluorescence staining was used to test the numbers of cleaved caspase-3-positive neurons.

*Experiment 3*: 30 SD rats were randomly divided into 3 groups: Sham, SAH+vehicle, and SAH+T0901317 (*n* = 10 per group). Rotarod test was performed at 7, 14, and 21 days. Morris water maze was performed on days 22-27 after SAH.

*Experiment 4*: to investigate the proposed molecular mechanism, 48 SD rats (*n* = 6 per group) were randomly divided into 7 groups: Sham, SAH+vehicle, SAH+T0901317, SAH+GSK2033+T901317, SAH+dimethyl sulfoxide (DMSO)+T0901317, SAH+IRF-1 CRISPR activation+T0901317, and SAH+scramble CRISPR+T0901317. Another 24 SD rats (*n* = 4 per group, except Sham group) were used for Terminal deoxynucleotidyl transferase dUTP nick end labeling (TUNEL) staining.

### 2.5. SAH Grade

SAH severity was blindly measured using the SAH grading score after sacrificed as previously described [21]. Only the rats sacrificed at 24 hours after modeling were used to collect SAH grade data. Briefly, the ventral side of rat brains was divided into 6 parts, and 0-3 score system was measured based on the volume of blood clot. The sum of six parts was calculated as the total score (0-18). Rats with SAH score under 7 were excluded.

### 2.6. Modified Garcia Score and Beam Balance Test

The short-term outcome was measured by modified Garcia score (a 3-18-point score system) and beam balance test (a 0-4-point score system) [21, 22]. The modified Garcia score is composed of six parts: spontaneous activity, spontaneous movement of all limbs, forelimbs outstretching, vibrissa touch, body proprioception, and climbing capacity. Beam balance score was evaluated base on the ability of animals walking on a wooden beam. The mean of three consecutive trials was calculated as the final score. Both scores with higher scores performed better neurological function.

### 2.7. Assessment of Long-Term Neurobehavioral Outcomes

The long-term outcome was measured by Rotarod test and Morris water maze test [23, 24]. The Rotarod test was performed at 7, 14, and 21 days post-SAH; the rats were placed on a rotating horizontal cylinder in limited wide lanes and were allowed to walk forward to avoid falling off the cylinder starting from 5 and 10 rpm, accelerated by 2 rpm every 5 seconds. The fallen time was recorded.

Water maze test was performed on 22-27 days post-SAH to evaluate animals' spatial learning ability and reference memory. The animals were trained 23-27 days, and on day 28, animals were tested for a probe trial without a platform. A video recording system traced the activities of animals and, the heatmap was recorded for quantification of distance, latency, and swimming speed by Video Tracking System SMART-2000 (San Diego Instrument Inc., CA, USA).

### 2.8. Western Blot

Western blot was performed as previously described [25]. Briefly, after euthanasia, rats were transcardially perfused with cold phosphate buffered saline (PBS 0.01 M, pH 7.4). Then, collect the brains, cut the left hemispheres, and snap frozen in liquid nitrogen. The samples were kept in -80°C until used. Brain samples were homogenized in RIPA lysis buffer (sc-24948, Santa Cruz Biotechnology Inc., TX, USA) and centrifuged at 14000 × g at 4°C for 30 min. Supernatant was collected and boiled with protein loading buffer 2x for 5 min. Equal amounts of protein (30 *μ*g) were loaded onto 10% SDS-PAGE gel, after electrophoresis and electrotransfer on 0.2 *μ*m nitrocellulose membranes. The membranes were blocked with 5% no-fat milk and incubated with the following primary antibodies at 4°C for overnight: anti-LXR-*α* (1 : 1000, ab176323, Abcam, MA, USA), anti-LXR-*β* (1 : 1000, ab56237, Abcam, MA, USA), anti-IRF-1 (1 : 1000, ab186384, Abcam, MA, USA), anti-PUMA (1 : 1000, ab33906, Abcam, MA, USA), anti-Drp1 (1 : 1000, ab184247, Abcam, MA, USA), anti-cleaved caspase-3 (1 : 500, 9661, Cell Signaling Technology Inc., MA, USA), anti-Bcl-2 (1 : 1000, ab59348, Abcam, MA, USA), anti-Bax (1 : 1000, ab32503, Abcam, MA, USA), and anti-GAPDH (1 : 2000, sc-365062, Santa Cruz Biotechnology Inc., TX, USA). Then, the membranes were incubated with the second antibodies (1 : 2000, Santa Cruz Biotechnology Inc., TX, USA) at room temperature for 2 hours. Immunoblots were detected by enhanced chemiluminescence (ECL) reagent kit (Amersham Bioscience, PA, USA) and quantified with optical methods using the ImageJ software (ImageJ 1.5, NIH, USA). The results were normalized using glyceraldehyde-3-phosphate dehydrogenase (GAPDH) as an internal control (1 : 2000, Santa Cruz Biotechnology Inc., TX, USA).

### 2.9. Immunofluorescence Measurement

Rats were transcardially perfused with cold PBS and 4% paraformaldehyde (PFA). The brains were kept and fixed in PFA at room temperature for 24 h before dehydration in 30% sucrose solution. Embed the brain in optimum cutting temperature (OCT) compound (Scigen Scientific Gardena, CA, USA) and frozen at -80°C. A cryostat was used to cut the brain into 10 *μ*m slides for the double immunofluorescence and TUNEL staining. The slides were observed and photographed with a fluorescence microscope (Lecia Microsystems, Germany).

Slides were washed with PBS three times, 10 min per time. Then, the slides were incubated with 3‰ Triton X-100 solution for 30 min and washed again with PBS for 3 times. Using 5% donkey serum to block the nonspecific binding, the slides were incubated at 4°C with primary antibodies: anti-LXR-*α* (1 : 100, ab176323, Abcam, MA, USA), anti-LXR-*β* (1 : 100, ab56237, Abcam, MA, USA), IRF-1 (1 : 1000, ab186384, Abcam, MA, USA), anti-PUMA (1 : 1000, ab33906, Abcam, MA, USA), anti-neuron-specific nuclear protein (NeuN) (1 : 100, ab177487, Abcam, MA, USA), and anti-cleaved caspase-3 (1 : 50, 9661, Cell Signaling Technology Inc., MA, USA). Then, the slides were washed with PBS and incubated with fluorescence-conjugated secondary antibodies (1 : 100, Jackson ImmunoResearch, PA, USA) for 2 hours at room temperature.

For TUNEL staining, double staining of neuron marker NeuN and TUNEL staining was performed with in situ Apoptosis Detection Kit (Roche, USA) at 24 h after SAH. The number of TUNEL-positive neurons was counted manually in the cortex. Six sections per brain were randomly chosen in 200x magnificent.

### 2.10. Statistical Analysis

Data were presented as mean ± standard deviation (SD) or median with interquartile range. The analyses were carried out using GraphPad Prism 7 (GraphPad Software Inc., San Diego, CA, United States) and SPSS (version 24.0; SPSS, Inc., Chicago, IL, United States). One-way analysis of variance (ANOVA) was used followed by Tukey's multiple comparison test to analyze differences among the groups, or long-term behavior, 2-way ANOVA, followed by Tukey's post hoc test, was used to compare the changes according to the different levels of multiple categorical variables. *p* value <0.05 was considered statistically significant.

## 3. Results

### 3.1. Mortality and SAH Grade

**Figure 1:**
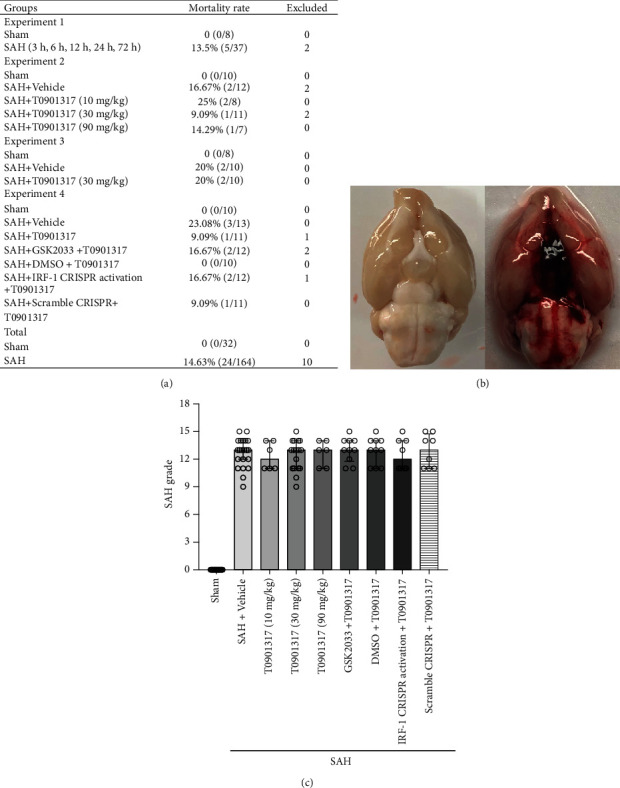
Mortality and subarachnoid hemorrhage (SAH) grade. (a) Animal usage and mortality of all experiment groups. (b) Representative pictures showed that subarachnoid blood clots were mainly presented around the circle of Willis in the rat brain at 24 h after SAH. (c) SAH grading scores of all SAH groups. Vehicle, 30% dimethyl sulfide (DMSO). Data were expressed as median ± 25th‐75th percentiles.

32 rats in Sham group and another 164 rats were subjected into SAH. There is no rat died in Sham group. Mortality of SAH rats was 14.72% (Figure 1(a)). After euthanasia, blood clots were present around the basal of brain (Figure 1(b)). There was no significant difference in average SAH grading scores among all SAH groups (Figure 1(c)).

### 3.2. Expression of LXR-*α*, LXR-*β*, and IRF-1

**Figure 2:**
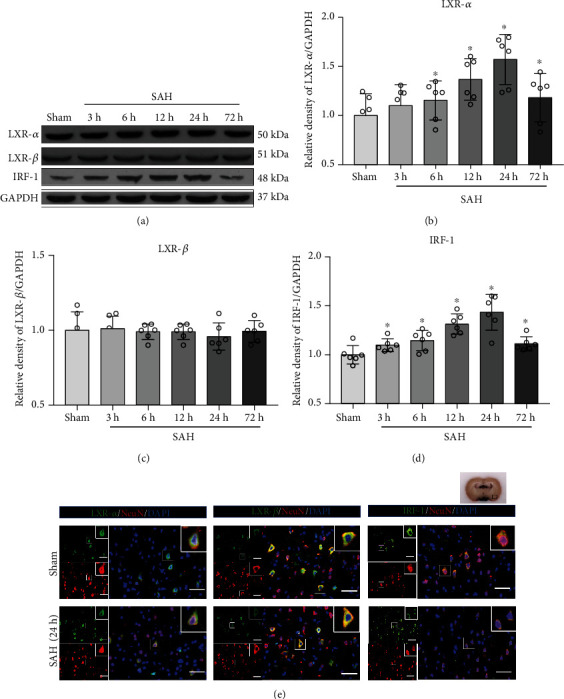
Time course and cellular colocalization of liver X receptor-*α* (LXR-*α*), liver X receptor-*β* (LXR-*β*), and interferon regulatory factor-1 (IRF-1). (a) Representative Western blot bands of time course and (b–d) quantitative analysis of LXR-*α*, LXR-*β*, and IRF-1. ^∗^*p* < 0.05 vs. Sham group. Error bars were presented with mean ± SD. *n* = 6 per group. (e) Representative double immunofluorescence staining for LXR-*α* (green), LXR-*β* (green), and IRF-1 (green) with neurons (NeuN, red) in the basal cortex of left hemisphere at 24 h after SAH. Nuclei were stained with DAPI (blue). *n* = 2 per group. Scale bar = 50 *μ*m.

Western blot was used to evaluate the expression of LXR-*α*, LXR-*β*, and IRF-1 at 3, 6, 12, 24, and 72 h after SAH (Figure 2(a)). The expression of LXR-*α* and IRF-1 significantly increased after SAH induction and reached the peak at 24 h when compared to Sham group, and then, it decreased to baseline at 72 h (*p* < 0.05; Figures 2(b) and 2(d)). The expression of LXR-*β* was unaffected after SAH induction (*p* < 0.05; Figure 2(c)). Immunofluorescence staining showed that LXR-*α*, LXR-*β*, and IRF-1 were localized in neurons (Figure 2(e)).

### 3.3. T0901317 Improved Neurobehavior and Attenuated Neuronal Apoptosis in Short-Term

**Figure 3:**
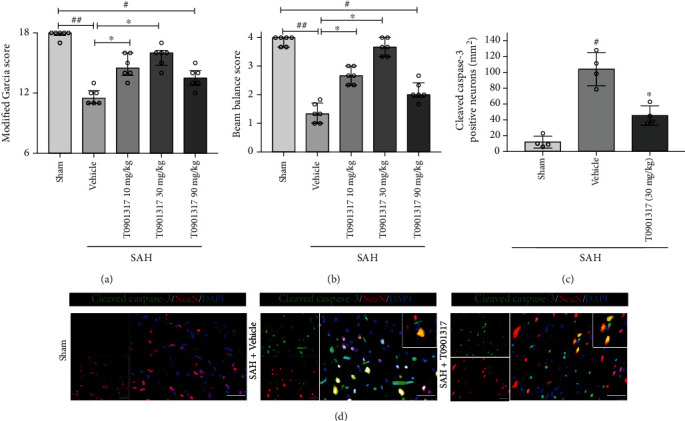
T0901317 attenuated the neurobehavior deficits and the number of cleaved caspase-3/neuron-specific nuclear protein- (NeuN-) positive cells at 24 h after subarachnoid hemorrhage (SAH). (a) Modified Garcia and (b) beam balance scores, *n* = 6 per group. Error bars were represented as median ± interquartile range using Kruskal-Wallis test followed by the Dunn post hoc test. (c) The quantification of double immunofluorescence staining. Error bars were represented with mean ± SD using one-way ANOVA followed by the Tukey post hoc test, *n* = 4 per group. (d) Representative double immunofluorescence staining of 3 groups. Scale bar = 50 *μ*m. ^#^*p* < 0.05 vs. Sham group; ^##^*p* < 0.01 vs. Sham group; ^∗^*p* < 0.05 vs. SAH+vehicle group. Vehicle, 30% dimethyl sulfide (DMSO).

Modified Garcia score and beam balance score were performed to test the short-term outcome. The results showed neurological function deficits in SAH+vehicle compared with Sham group (*p* < 0.05; Figures 3(a) and 3(b)). Treatment with 30 mg/kg of T0901317 significantly improved the neurological scores the most among three different dosage groups (*p* < 0.05, Figures 3(a) and 3(b)). Therefore, 30 mg/kg was chosen for further experiments.

Double immunofluorescence staining of cleaved caspase-3 and NeuN showed in cortex that the number of cleaved caspase-3-positive neurons in SAH+vehicle group is larger than that in Sham group (*p* < 0.05, Figures 3(c) and 3(d)); the treatment of T0901317 can significantly decrease the number of cleaved caspase-3-positive neurons compared with SAH+vehicle group (*p* < 0.05, Figures 3(c) and 3(d)).

### 3.4. T0901317 Improved Neurobehavior and Reduced Hippocampal Neuronopathy in Long-Term

**Figure 4:**
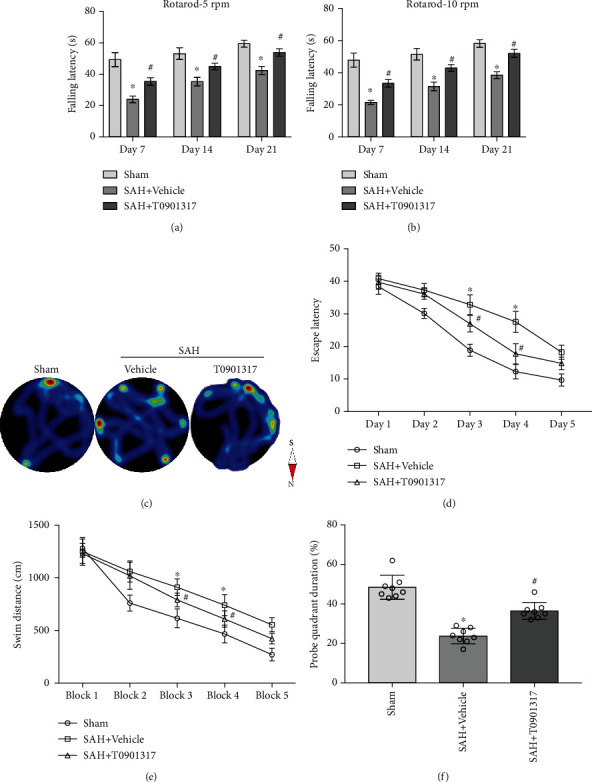
T0901317 attenuated long-term neurological deficits after subarachnoid hemorrhage (SAH). Rotarod test of (a) 5 rpm and (b) 10 rpm on days 7, 14, and 21 post-SAH. (c) Representative imaging pictures of heatmap in the probe trial. (d, e) Quantification of escape latency and swimming distance of Morris water maze test from days 22 to 26. (f) Quantification of probe quadrant duration in probe trial on day 27 post-SAH. *n* = 8 per group. Data were expressed as the means ± SD using one-way ANOVA followed by the Tukey post hoc test. ^∗^*p* < 0.05 vs. Sham group; ^#^*p* < 0.05 vs. SAH+vehicle group. Vehicle, 30% dimethyl sulfide (DMSO).

Rotarod test results showed a significant shorter falling latency in SAH+vehicle group compared with Sham group in 5 revolutions per minute (rpm) and 10 rpm (*p* < 0.05, Figures 4(a) and 4(b)). The treatment of T0901317 reversed the poor performance compared with SAH+vehicle in both 5 and 10 rpm (*p* < 0.05, Figures 4(a) and 4(b)).

Water maze test showed the spatial memory loss in SAH+vehicle group compared with Sham group in escape latency and distance to find the platform; the treatment of T0901317 significantly improved the performance at blocks 3 and 4 (*p* < 0.05, Figures 4(c)–4(e)). In probe quadrant trial, SAH+vehicle group spent less time in target probe quadrant compared with Sham group; the treatment of T0901317 significantly improved the spent time in target probe quadrant compared with SAH+vehicle (*p* < 0.05, Figure 4(f)).

### 3.5. GSK2033 Reversed the Neuroprotective Effect of T0901317 in Neuronal Apoptosis

**Figure 5:**
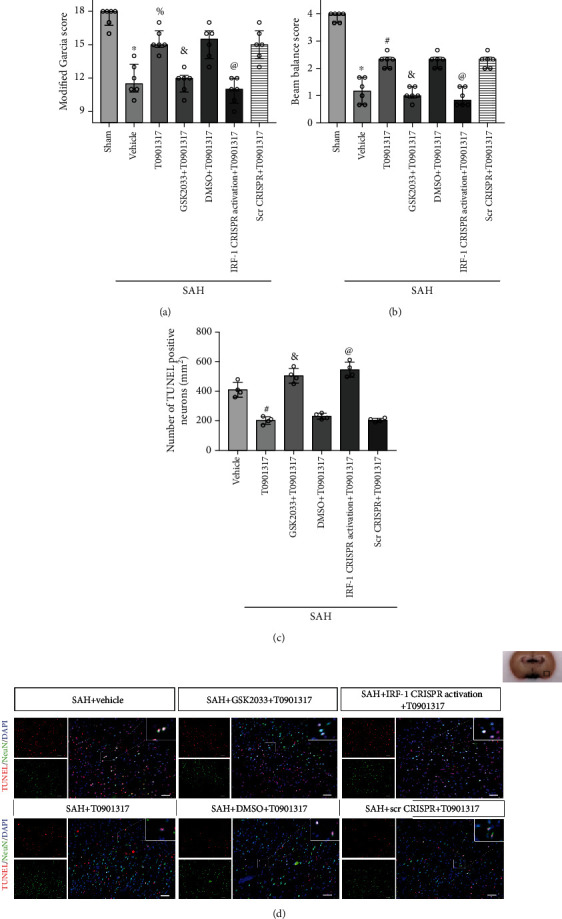
T0901317 attenuated neurological deficits and the number of apoptosis neurons, which were abolished by GSK2033 and IRF-1 CRISPR activation. (a) Modified Garcia and (b) beam balance scores, *n* = 6 per group. Error bars were represented as median with interquartile range using Kruskal-Wallis test followed by the Dunn post hoc test. (c) The quantification of TUNEL staining. Error bars were represented with mean ± SD using one-way ANOVA followed by the Tukey post hoc test, *n* = 4 per group. (d) TUNEL staining of all 6 groups. Scale bar = 50 *μ*m. ^∗^*p* < 0.05 vs. Sham group; ^#^*p* < 0.05 vs. SAH+vehicle group; ^&^*p* < 0.05 vs. SAH+DMSO+T0901317 group; ^@^*p* < 0.05 vs. SAH+Scr CRISPR+T0901317 group. Scr CRISPR: scramble CRISPR. Vehicle, 30% dimethyl sulfide (DMSO).

**Figure 6:**
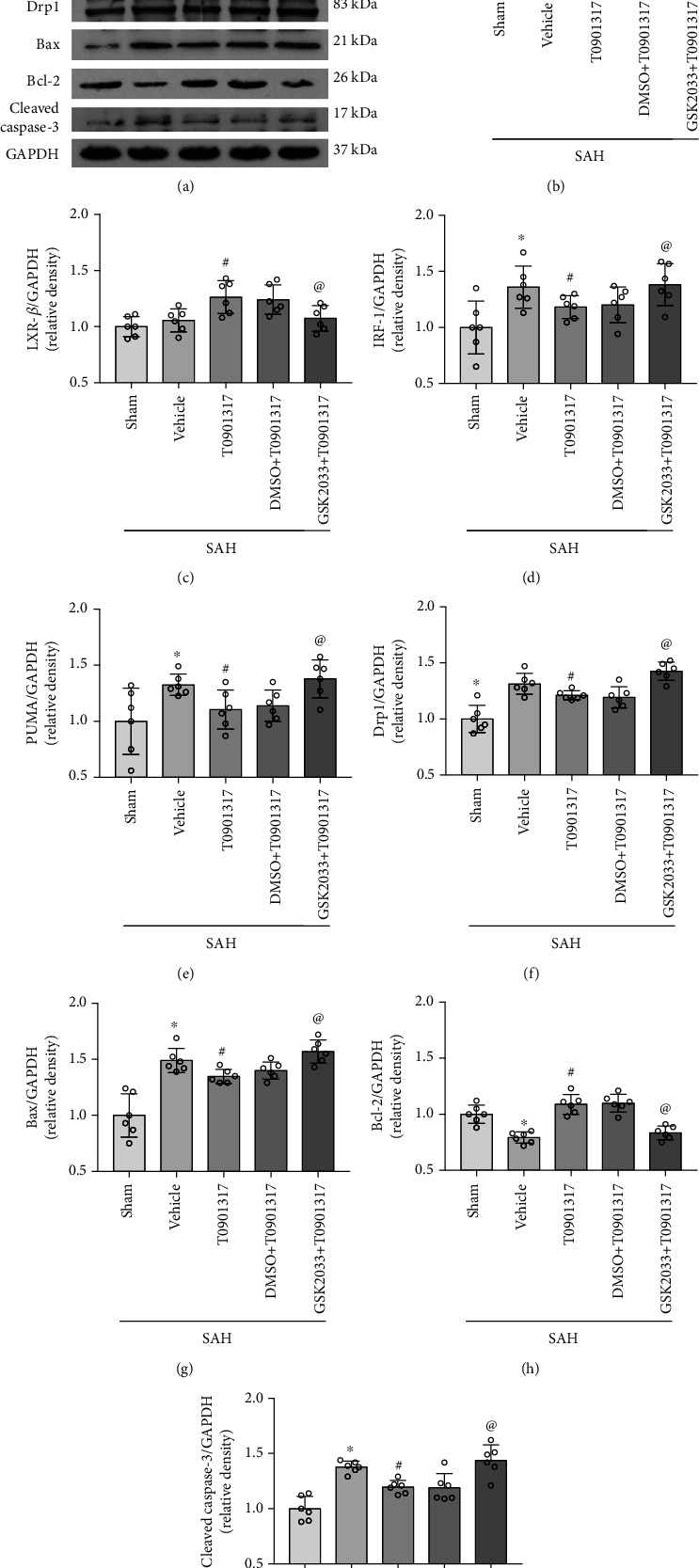
GSK2033 abolished the antiapoptotic effect of T0901317 at 24 h after subarachnoid hemorrhage (SAH). (a) Representative Western blot bands. (b–i) Quantification of LXR-*α*, LXR-*β*, IRF-1, PUMA, Drp1, Bcl-2, Bax, and cleaved caspase-3 in the ipsilateral hemisphere at 24 h after SAH. Data was represented as mean ± SD. ^∗^*p* < 0.05 vs. Sham group, ^@^*p* < 0.05 vs. SAH+vehicle group, and ^#^*p* < 0.05 vs. SAH+DMSO+T0901317 group; One-way ANOVA and Tukey's post hoc test, *n* = 6 per group. Vehicle, 30% dimethyl sulfide (DMSO).

GSK2033 abolished effects of T0901317 in SAH rats. Compared with SAH+DMSO+T0901317 group, GSK2033 abolished the protective effects of T0901317 both in modified Garcia and beam balance results in SAH+GSK2033+T0901317 group (*p* < 0.05, Figures 5(a) and 5(b)). Western blot results showed a significant increase in the expression of LXR-*α*, IRF-1, PUMA, Drp1, Bax, and cleaved caspase-3 and a decrease in Bcl-2 in SAH+vehicle group compared with Sham group (*p* < 0.05, Figures 6(a), 6(b), and 3(d)–3(i)). The treatment of T0901317 significantly decreased the expression of IRF-1, PUMA, Drp1, Bax, and cleaved caspase-3 and increased the expression of LXR-*α* and Bcl-2 compared with SAH+vehicle group (*p* < 0.05, Figures 6(a), 6(b), and 6(d)–6(i)). There was no significant difference in the expression of LXR-*β* among Sham, SAH+vehicle, and SAH+T0901317 group (*p* < 0.05, Figure 6(c)). Inhibition of LXRs with GSK2033 abolished effects of T0901317 with a decrease of LXR-*α*, LXR-*β*, and Bcl-2 and an increase of IRF-1, PUMA, Drp1, Bax, and cleaved caspase-3 in SAH+GSK2033+T0901317 group, compared with SAH+DMSO+T0901317 group (*p* < 0.05, Figures 6(a), 6(b), and 6(d)–6(i)).

TUNEL staining results showed that the number of apoptotic neurons increased in SAH+vehicle group, compared with Sham group (*p* < 0.05, Figures 5(c) and 5(d)), while the treatment of T0901317 decreased the number of apoptotic neurons compared with SAH+vehicle group (*p* < 0.05, Figures 5(c) and 5(d)). In SAH+GSK2033+T0901317 group, the number of apoptotic neurons increased compared with SAH+DMSO+T0901317 group (*p* < 0.05, Figures 5(c) and 5(d)).

### 3.6. IRF-1 CRISPR Activation Abolished the Antiapoptotic Effect of T0901317 in SAH

**Figure 7:**
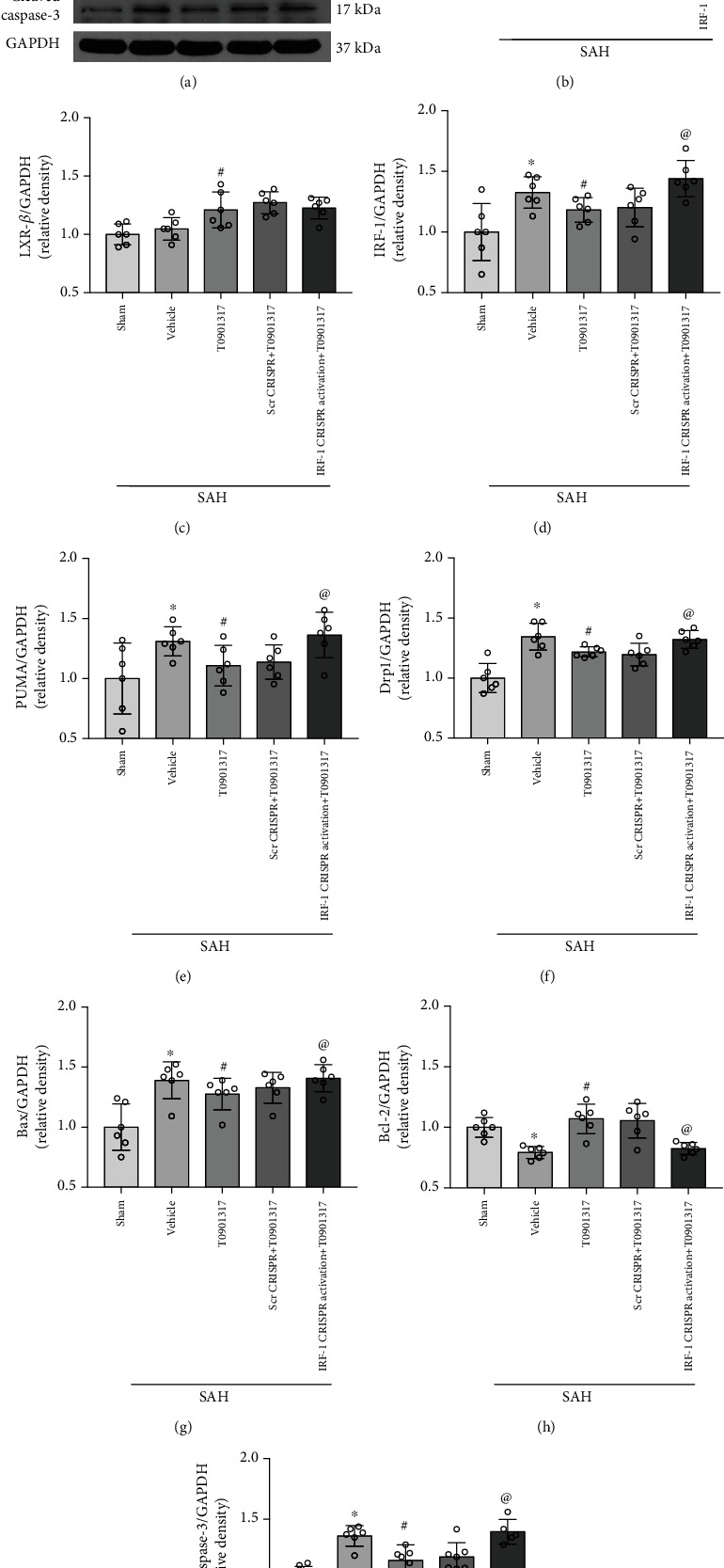
IRF-1 CRISPR activation abolished the antiapoptotic effect of T0901317 at 24 h after subarachnoid hemorrhage (SAH). (a) Representative Western blot bands. (b–i) Quantification of LXR-*α*, LXR-*β*, IRF-1, PUMA, Drp1, Bcl-2, Bax, and cleaved caspase-3 in the ipsilateral hemisphere at 24 h after SAH. Data was represented as mean ± SD. ^∗^*p* < 0.05 vs. Sham group, ^@^*p* < 0.05 vs. SAH+vehicle group, and ^#^*p* < 0.05 vs. SAH+Scr CRISPR+T0901317 group; One-way ANOVA and Tukey's post hoc test, *n* = 6 per group. Scr CRISPR: scramble CRISPR. Vehicle, 30% dimethyl sulfide (DMSO).

Compared with SAH+scramble CRISPR+T0901317 group, IRF-1 CRISPR activation abolished the protective effects of T0901317 both in modified Garcia and beam balance in SAH+IRF-1 CRISPR activation+T0901317 group (*p* < 0.05, Figures 5(a) and 5(b)). Western blot results revealed a significant increase in the expression of IRF-1, PUMA, Drp1, Bax, and cleaved caspase-3 and a decrease in Bcl-2 in SAH+vehicle group compared with Sham group (*p* < 0.05, Figures 7(a) and 7(d)–7(i)). IRF-1 CRISPR activation abolished effects of T0901317 with a significant increase of IRF-1, PUMA, Drp1, Bax, and cleaved caspase-3 and a decrease of cleaved caspase-3 in SAH+IRF-1 CRISPR activation+T0901317 group compared with SAH+scramble CRISPR+T0901317 group (*p* < 0.05, Figures 7(a) and 7(d)–7(i)).

TUNEL staining results showed that the number of apoptosis neurons increased in SAH+IRF-1 CRISPR activation+T0901317 group, compared with SAH+scramble CRISPR+T0901317 group (*p* < 0.05, Figures 5(a) and 5(d)).

## 4. Discussion

In the present study, our results revealed that there was an increase in expression of LXR-*α* and IRF-1, and there were no changes in LXR-*β* at 24 h post-SAH. LXR-*α*, LXR-*β*, and IRF-1 were expressed in neurons. The treatment of T0901317 improved in both short- and long-term neurological impairment and attenuated neuronal apoptosis. Inhibition of LXRs with GSK2033 and IRF-1 CRISPR activation abolished the neuroprotective effects of T0901317, which was related with the overexpression of IRF-1, PUMA, and Bcl-2 and downregulation of Drp1, Bax, and cleaved caspase-3. IRF-1 CRISPR activation abolished the neuroprotective effect of T0901317 with an increase of IRF-1 and PUMA and downstream Drp1, Bax, and cleaved caspase-3, but did not affect LXRs. Taken together, the results suggest that T0901317 attenuated the neurological impairment and neuronal apoptosis, possibly at least via LXRs/IRF-1/PUMA/Drp1 pathway after SAH.

While the brain only comprises 2.5% of the total body mass on average, it contains about 23% of the total cholesterol, indicating that cholesterol or its associated receptors play important roles in brain function [26]. LXRs are two members of nuclear receptors that modulate the cholesterol and were reported to be neuroprotective in CNS disease [27]. LXR-*α* is highly expressed in the liver, adipose tissue, adrenal glands, intestine, kidney, and macrophages, while LXR-*β* is widely expressed in whole body [7]. Our results showed both LXR-*α* and LXR-*β* were detected in SAH rat brains, which is agreement with previous reports that both LXRs were detected in rats' brain [28]. It was reported that LXRs were activated in several pathological processes, such as ischemia/reperfusion injury, AD, and PD [29–31]. But our results indicated that LXR-*α* was enhanced in EBI after SAH, while LXR-*β* remained unaffected. These results were in agreement of that was reported in ICH [6]. The reasons may be determined by the expression and function of LXR-*β*. Firstly, LXR-*β*'s expression was higher in brain and relatively stable [32, 33]. Secondly, LXR-*β* was reported to play important roles in guiding the migration of neurons, protecting dopaminergic neurons in PD [30, 34]. Some studies have revealed that activating both LXR-*α* and LXR-*β* can inhibit inflammation [35]. It is reported that LXR-*α* activation can inhibit the apoptosis of macrophage, breast cancer cell, or prostate cancer cells [36–38]. In our study, we got the similar results that LXR activation with synthetic agonist (T0901317) can decrease the apoptosis neurons.

T0901317 is a synthetic agonist for LXRs used to observe the expression of all dietary cholesterol-regulated genes in many models [39]. The treatment of T0901317 as agonist of LXRs was reported to provide a potential therapy for ischemia stroke, AD, PD, and ICH [6, 8, 11, 40, 41]. However, there is no research published to evaluate T0901317 as a treatment of SAH. In this research, we have discovered that T0901317 has the ability to attenuate neuronal apoptosis during both short- and long-term after SAH. We have established a reasonable mechanism for how T0901317 activating LXRs improved neurological function. What is more, T0901317 can easily penetrate the blood-brain barrier, which is more suitable for clinical use. We chose 3 different dosages in our investigation of treatment [6], and our results suggest that 30 mg/kg of T0901317 is most effective for neurological recovery in experimental SAH of rats.

The present study revealed that the expression of IRF-1 was enhanced in the left-brain hemisphere and peaked at 24 h post-SAH. These results were similar with the previous reports showing IRF-1 played an important role in ischemic neuronal death in mice or dead ischemic patients [15]. IRF-1 was also reported to aggravate hepatic ischemic/reperfusion injury [42]. Our results also showed that activating LXRs inhibited the expression of IRF-1 on transcription level, which was in agreement with previous reports showing that LXR ligands inhibit neither STAT1 phosphorylation nor STAT1 translocation to the nucleus but rather inhibit STAT1 binding to the promoters and the expression of IRF-1 [17]. Immunoprecipitation data revealed that LXR-*β* formed a trimer with PIAS1-pSTAT1, whereas LXR-*α* formed a trimer with HDAC4-pSTAT1, mediated by direct ligand binding to the LXR proteins [17]. In our study, inhibiting IRF-1 decreased the expression of PUMA, which was in agreement with the reports showing that IRF-1 transcriptionally upregulated PUMA [18]. PUMA was reported to mediate the mitochondrial apoptotic pathway in IRF-1-induced apoptosis in gastric and breast cancer cells [18]. It is notable that in the PUMA promoter, there appears to be an IRF-1-binding site [18]. Both PUMA and Drp1 are quite important in maintaining mitochondrial stability in various disorders of central nervous system, including AD, PD, Huntington disease, acute spinal cord injury, and traumatic brain injury [43–47]. It was reported that activation of PUMA would trigger the recruitment of a type of Drp1 to mitochondrial membrane and caused release of cytochrome c, again trigging the apoptotic cascades [20, 48]. Our results showed that activation of LXRs mediated the expression of IRF-1 and its downstream PUMA, Drp1, Bcl-2, Bax, and cleaved caspase-3.

The results of our investigation support treatment of T0901317 as a therapy to attenuate neuronal apoptosis. However, there are limitations. First, although we tested both subtypes of LXRs, we still cannot decide which is more important in preventing neuronal apoptosis for the lack of selective agonist or antagonist. Second, because there is no stable in vitro model of SAH, our hypothesis mechanism was only tested in vivo model. In addition, LXRs are reported to inhibit neuroinflammations in many CNS diseases, and our experiment design cannot exclude the benefit of T0901317's anti-inflammation effect. All of above need to be further investigated.

## 5. Conclusion

We demonstrated that T0901317 improved in both short- and long-term neurological impairment and attenuated neuronal apoptosis partly through LXRs/IRF-1/PUMA/Drp1 signaling pathway in EBI after SAH. Thus, we may provide a new therapeutic treatment against EBI.

## Data Availability

The data support the findings of this study are available from the corresponding author upon reasonable request.
